# Midwives’ perceptions of and attitudes towards prevention of mother-to-child-transmission of HIV

**DOI:** 10.4102/curationis.v46i1.2353

**Published:** 2023-06-15

**Authors:** Rita Opoku-Danso, Debbie S.K. Habedi

**Affiliations:** 1 Department of Health Studies, Faculty of Nursing, University of South Africa, Pretoria, South Africa; 2 Department of Health Studies, Faculty of Public Health, University of South Africa, Pretoria, South Africa

**Keywords:** attitudes, human immunodeficiency virus (HIV), midwives, perceptions, prevention of mother-to-child transmission (PMTCT)

## Abstract

**Background:**

Prevention of mother-to-child transmission (PMTCT) of HIV services has become an integral part of antenatal services. Prevention of mother-to-child transmission was introduced in all the regions of Ghana, but mother-to-child transmission (MTCT) continued to increase.

**Objectives:**

To explore and describe midwives’ perceptions and attitudes towards PMTCT of HIV services.

**Method:**

Quantitative research approach and descriptive cross-sectional design were used. The population includes all midwives between the ages of 21 and 60 years who work in antenatal care (ANC) clinics in 11 district hospitals in the Central Region of Ghana where the study was conducted. Forty-eight midwives were interviewed using a census sample process. Data were analysed using the Statistical Package for the Social Sciences version 21. Correlation analysis was performed to find the relationships between the attitudes and the perceptions of the midwives on PMTCT of HIV services.

**Results:**

Seventy percent of midwives had positive perceptions of PMTCT of HIV services and 85% had positive attitudes towards the provision of PMTCT of HIV services. Midwives were screening all pregnant women who visited the ANCs and referring those who tested positive to other institutions where they can be monitored. Some of the concerns considered were views on retesting HIV-infected pregnant women throughout their pregnancy. There was a positive correlation between attitudes and perceptions of midwives on PMTCT of HIV services.

**Conclusion:**

Midwives had positive perceptions and positive attitudes towards the PMTCT of HIV services that they were providing to antenatal attendees. Also, as the attitudes of the midwives towards PMTCT of HIV services improved, their perceptions of PMTCT services also improved.

**Contribution:**

Decentralisation of PMTCT of HIV services to community-based health facilities is appropriate to enable sub-district health facilities to test for HIV and provide counselling services to pregnant women.

## Introduction

Approximately 38 million people as of 2022 were living with human immunodeficiency virus (HIV), and tens of millions of people have died of acquired immunodeficiency syndrome (AIDS)-related causes since the beginning of the epidemic (kff.org 2023). However, there are persistent renewed efforts and commitments to halt and reverse the HIV pandemic. This has been seen in efforts such as the Sustainable Development Goal (SDG-3) which aims at ensuring universal access to HIV prevention services and to end HIV as a global threat by the year 2030 (Meilani, Setiyawati & Barasa 2019:88). Despite effective prevention of mother-to-child transmission (PMTCT) given to mothers and their children by first being given information about methods to avoid HIV/AIDS (Hutagaol 2022:26), worldwide the epidemic is increasing and infecting millions of people every year.

According to Choi et al. (2021:1), utilisation of antenatal care services in Ghana has substantially increased over the years, but the rates of mother-to-child transmission (MTCT) of HIV are still high. Not all pregnant women were informed prior to testing nor informed of their test results. Many mothers indicated that pretest counselling is limited, although the midwives claimed to provide it. This study aimed to explore and describe midwives’ perceptions and attitudes towards PMTCT of HIV services in the Central Region of Ghana.

## Literature review

In sub-Saharan Africa, where nearly two-thirds of the world’s diseases live, 55% of adults living with HIV are women than victims (UNAIDS 2021:2). As the number of women infected with the virus increased, so did the number of children infected through their mothers (UNAIDS 2021:2). About 1800 new HIV infections occur every day in children under 15 years of age, and more than 90% of them occur in developing countries. Most of these infections (nearly 90%) are associated with MTCT (UNAIDS 2021:2).

Rowan et al. (2018:1) alluded that without treatment during pregnancy, HIV positive mothers have up to a 45% chance of transmitting the infection to their children. With appropriate treatment, such as that delivered through PMTCT programmes, these odds can be reduced to nearly zero.

Unfortunately, due to a variety of challenges in PMTCT programme implementation, PMTCT programmatic success in many countries remains suboptimal (Rowan et al. 2018:1). Vertical transmission (mother-to-child) is a major pathway for HIV in children. The World Health Organization (WHO) has declared that without preventive interventions, 40% of babies born from infected mothers would themselves be infected. The focus of prevention is applied in three phases, namely pregnancy, labour and breastfeeding. These three underscore the critical midwives’ roles that play in PMTCT. Midwives’ knowledge of HIV can be the foundation that influences people’s attitude and behaviour. However, there are still midwives with negative attitude and perception towards HIV/AIDS. Thus, it is paramount that it becomes part of their job to overcome HIV/AIDS with unique understanding and approach. Therefore, midwives’ knowledge, attitude and perception towards HIV are important determinants in overcoming the disease (Meilani et al. 2019:89).

## The research method and design

### Study design

This study used a descriptive cross-sectional study design and quantitative research methods to explore and describe midwives’ perceptions and attitudes towards PMTCT of HIV services in the Central Region of Ghana. A close-ended questionnaire was used, which helped the researchers to identify a general pattern of respondents’ reactions to the PMTCT of HIV services. This was used to achieve a comprehensive and generalisable set of results that were presented in a concise and meaningful manner (Sharma 2018:4).

### Setting

The survey was conducted in 1 of the 16 regions along the coast of Ghana called the Central Region. With the historic city of Cape Coast as the capital, there are 20 administrative districts in the region. Eleven of the 20 districts have district hospitals serving the region and are the focus of our research. These 11 district hospitals also have well-organised antenatal clinics (ANCs), which include PMTCT of HIV services as part of its activities.

### Study population and sampling procedure

The study population included all midwives at the ANCs who were employed from January 2019 to December 2019 at the 11 district hospitals in the Central Region of Ghana. A census sampling procedure was used to select all 48 midwives (Table 1). Hence, the total number of midwives in 11 local hospitals was 48. Since each district hospital already had the number of midwives in the ANCs, the same numbers in the district hospitals were used for the distribution of the questionnaires. The contact details of the midwives were obtained from the administrators of the selected district hospitals, which included information regarding places of residence, phone numbers and other contact details that could be obtained to ensure contact with them when necessary.

**TABLE 1:** Population of midwives at the antenatal clinics across district hospitals in the Central Region during the data collection.

District hospitals	Population of midwives
Abura Dunkwa Hospital	3
Ajumako District Hospital	3
Cape Coast Metropolitan Hospital	6
Dunkwa Municipal Hospital	5
St. Francis Xavier Hospital	4
St. Luke Catholic Hospital	3
Swedru Government Hospital	6
Praso Government Hospital	6
Saltpond Hospital	4
Our Lady of Grace Hospital	4
Winneba Municipal Hospital	4

**Total**	**48**

The midwives were recruited through one-on-one contact, during which we explained to them the purpose of the study and the need to respond by completing the questionnaires. This was done by providing the respondent information sheet (RIS) for all the midwives to read and understand. After they had read and understood the RIS, they were given a consent form to sign before the questionnaires were implemented. Permission to access personally identified information was also sought.

The midwives were selected to meet the inclusion criteria, which means that all midwives working at the ANCs of the selected district hospitals must be between the ages of 21 and 60 years. The study did not include midwives who do not work at the ANCs, those who have retired or working on contract basis, as well as rotation midwives and student midwives.

### Validity and reliability of data collection instrument

Pretesting refers specifically to refining the questionnaire from sampling and recruitment strategies through data collection and analysis. Furthermore, it can assess the cognitive process of answering questions to increase question validity (Warddropper et al. 2020:1650). This was conducted on 05 January 2020, with two midwives each at the University of Cape Coast Hospital and the University of Education Winneba Municipal Hospital. In this study, the principal component factor analysis was used to measure content validity and any question that scored 0.3 and below was considered irrelevant and removed. The responses from the pretesting were used to modify the instruments. Feedback from the supervisor, University of South Africa (UNISA) Health Studies Research Ethics Committee and Ghana Health Services Ethics Review Committee was used to improve the instrument’s reliability.

### Data collection method

The researchers administered questionnaires to respondents after obtaining consent, and ANC data were collected only from midwives who were on duty, not representative of midwives who were on leave. The purpose and objectives of the study were well explained to the respondents to achieve their full participation and allow them to make an informed decision. Data collection began on 01 April 2020 and continued until 03 June 2020, during which time all coronavirus disease 2019 (COVID-19) protocols were strictly adhered to. The basic preventive measures for protecting the researcher and respondents were implemented as:

- wearing of face masks
- maintaining social distancing of 1.5 m
- using hands sanitizers for hands hygiene

The aforementioned preventive measures were also followed during distribution of the questionnaires.

Excel was used to capture the data and the Statistical Package for the Social Sciences (SPSS) version 21 was used for statistical analysis.

The designing of labels was done for identification of variables that could be examined. Descriptive statistics and inferential statistics were used in analysing the data. The descriptive statistics analysis was used to compute frequencies, percentages, means and standard deviation for the independent and dependent variables, while the inferential statistics analysis was used to describe the respondents’ perceptions and attitudes towards PMTCT of HIV services. Questionnaires were distributed to midwives in the various ANCs in the 11 district hospitals within the region.

### Ethical considerations

Ethical approval was obtained from the Health Studies Research Ethics Committee (HSREC) of the Department of Health Studies, University of South Africa. (No. HSHDC/896/2019). In addition, permission was obtained from the Regional Health Directorate in the Central Region of Ghana, the district hospitals in the region, as well as the midwives of the various ANCs at the selected district hospitals.

The researchers sought the consent of the respondents before giving them the research instruments. The respondents were informed about the type of questions to expect in the questionnaire and the purpose of the study. They were also ensured that their responses would remain anonymous. The respondents were also assured of their right to withdraw at any time.

## Results

### Demographic characteristics

The age of the respondents ranged from 21 to 30 years (47.9%), 31 to 40 years (37.5%), 41 to 50 years (10.4%) and 51 to 60 years (4.2%). The mean age was 33.8 years, with a standard deviation of 6.3. Most of the respondents, 34 (70.8%), had a degree in midwifery, while 14 (29.2%) had a diploma in midwifery.

Twenty-one of the sampled midwives (43.8%) were staff midwives, 11 (22.9%) were senior midwifery officers, 7 (14.6%) were senior staff midwives and 8 (16.7%) were midwifery officers. Total number of respondents was 48. All the survey questionnaires were completed except for only one sampled midwife. Twenty-three of the sampled midwives (47.9%) had worked for between 1 and 3 years, whereas 9 (18.8%) had worked as midwives for between 4 and 5 years, 7 (14.6%) had worked for between 6 and 9 years and another 9 (18.8%) had worked for 10 years or more. The mean number of years the respondents had worked as midwives was 3.8, with a standard deviation of 0.71, which showed that the sampled midwives from the 11 district hospitals had provided PMTCT of HIV services to antenatal attendees over many years, which could have contributed to the development of their skills, competencies and experience. The many years that the respondents functioned as midwives also enabled them to provide valuable information about their assessment of the PMTCT of HIV services.

### Perceptions of midwives on prevention of mother-to-child transmission of HIV services

The study explored and described the perceptions of midwives on PMTCT of HIV services in the Central Region of Ghana. This was important because Meilani et al. (2019:90) argued that midwives’ perceptions and understanding of PMTCT services influenced their behaviour and interactions with HIV-positive pregnant women.

Some of the issues considered in the study were the mothers’ lack of knowledge of HIV and PMTCT of HIV services, the scarcity of infrastructure for PMTCT of HIV services, CD4 count levels’ hindering access to the eligibility of government treatment, as well as insufficient voluntary and counselling centers.

### Some mothers lack knowledge of HIV and prevention of mother-to-child transmission services

Table 2 shows that the majority (79.1%) of the midwives strongly agreed (33.3%) and agreed (45.8%) that some mothers lack knowledge of HIV and PMTCT services. This implies that most of the midwives conceded that some mothers lack knowledge of HIV and PMTCT services in the various district hospitals. This is likely to affect the effectiveness of PMTCT of HIV services in the region, as some mothers may be late in ANC reporting to the designated health facilities.

**TABLE 2:** Perceptions of midwives on prevention of mother-to-child transmission of HIV service administration.

Perceptions	Responses	Frequency	Percentage (%)
Some mothers lack knowledge of HIV and PMTCT.	Strongly agree	16	33.3
	Agree	22	45.8
	Disagree	6	12.5
	Strongly disagree	3	6.3
	Don’t know	1	2.1
	Total	48	100.0
There is a scarcity of infrastructure for PMTCT of HIV services.	Strongly agree	17	35.4
	Agree	15	31.3
	Disagree	10	20.8
	Strongly disagree	6	12.5
	Total	48	100.0
The CD4 count level hinders access to the eligibility of government treatment.	Strongly agree	7	14.6
	Agree	7	14.6
	Disagree	12	25.0
	Strongly disagree	17	35.4
	Don’t know	5	10.4
	Total	48	100.0
Pregnant teens lack self-care knowledge.	Strongly agree	21	43.8
	Agree	25	52.1
	Strongly disagree	2	4.2
	Total	48	100.0
The midwives lack adequate PMTCT of HIV training.	Strongly agree	8	16.7
	Agree	10	20.8
	Disagree	10	20.8
	Strongly disagree	20	41.7
	Total	48	100.0
The hospital has a shortage of ARV drugs.	Strongly agree	6	12.5
	Agree	7	14.6
	Disagree	26	54.2
	Strongly disagree	9	18.8
	Total	48	100.0
Some religions do not condone visits to health facilities.	Strongly agree	13	27.1
	Agree	10	20.8
	Disagree	14	29.2
	Strongly disagree	8	16.7
	Don’t know	3	6.3
	Total	48	100.0
Male partners need to give consent before pregnant women can be tested for HIV.	Strongly agree	14	29.2
	Agree	13	27.1
	Disagree	5	10.4
	Strongly disagree	16	33.3
	Total	48	100.0
There are not enough midwives at the ANCs.	Strongly agree	12	25.0
	Agree	17	35.4
	Disagree	10	20.8
	Strongly disagree	8	16.7
	Don’t know	1	2.1
	Total	48	100.0
Some religions do not condone condom use.	Strongly agree	25	52.1
	Agree	20	41.7
	Disagree	2	4.2
	Strongly disagree	1	2.1
	Total	48	100.0
The hospitals have a shortage of HIV testing kits.	Strongly agree	13	27.1
	Agree	21	43.8
	Disagree	9	18.8
	Strongly disagree	4	8.3
	Don’t know	1	2.1
	Total	48	100.0

ARV, Antiretroviral; ANC, antenatal clinic; PMTCT, prevention of mother-to-child transmission.

### There is shortage of infrastructure for prevention of mother-to-child transmission services

Majority (66.7%) of the midwives strongly agreed (35.4%) and agreed (31.3%) that there was a shortage of infrastructure for PMTCT of HIV services in the designated hospitals. This denotes that the majority of the midwives believed that a shortage of infrastructure for PMTCT of HIV services in the designated hospitals could seriously affect the quality of PMTCT of HIV services to be provided by the midwives at these hospitals.

### The CD4 count level hinders antiretroviral treatment

Majority (60.4%) of the midwives disagreed (25%) and strongly disagreed (35.4%) that CD4 count levels hinder access to the eligibility of Ghana Government treatment. This implies that majority of the midwives rejected the idea that CD4 count levels hindered the access of some HIV-infected pregnant women to the eligibility of government treatment under the PMTCT of HIV services in the region. This means that antiretroviral therapy (ART) is initiated irrespective of the CD4 cell count level. However, 17 of the midwives (29.6%) believed that CD4 count levels impede eligibility of government treatment, which was dangerous, because HIV-positive pregnant women who have been cared for by these midwives could infect their unborn children before the start of ART.

### Pregnant teens lack self-care knowledge

The study found that most (95.9%) of the midwives strongly agreed (43.8%) and agreed (52.1%) that pregnant teens lack self-care knowledge. This means that most of the midwives believe that pregnant teens attending ANCs in the region lack self-care knowledge and therefore cannot care for themselves or their unborn babies while receiving PMTCT of HIV services. Only two (4.2%) of the midwives disagreed with this statement.

### Some religions do not accept condom use

More than half (52.1%) of the midwives strongly agreed that some religions do not condone condom use and a little less than half (41.7%) of the midwives agreed with that viewpoint. This means that the majority of the midwives (93.9%) believe that some religions do not approve of condom use, which contributes to HIV infection and pregnancy among girls and women in the region (see Table 2).

### The midwives lack adequate prevention of mother-to-child transmission of HIV training

The results presented in Table 2 showed that the majority (62.5%) of the midwives disagreed (20.8%) and strongly disagreed (41.7%) that midwives lack adequate PMTCT of HIV training. This implies that the perception that midwives lack adequate PMTCT of HIV training to provide the needed care and support to HIV patients was disputed by most of the respondents.

### The hospital has a shortage of antiretroviral drugs

More than half (54.2%) of the midwives disagreed that the hospitals had a shortage of antiretroviral (ARV) drugs and less than one-third of them (18.8%) strongly disagreed with that opinion (see Table 2). This means that the majority of midwives (73%) rejected the idea of a shortage of ARV drugs in the hospitals.

### Some religions do not accept visits to health facilities

Just over a quarter of the midwives (27.1%) strongly agreed that some religions do not condone visiting health facilities and just under a quarter (20.8%) agreed. This implies that slightly less than half (47.9%) of the midwives believe that some religions do not condone visits to health facilities. This could have a negative impact on PMTCT of HIV services in the region, as some HIV-positive women may report to health facilities at an advanced stage of infection.

### Male partners need to give consent before pregnant women may be tested for HIV

Majority of the midwives (56.3%) strongly agreed (29.2%) and agreed (27.1%) that consent of the male partner is required to test pregnant women for HIV. This means that most midwives (83.4%) believe that consent of the male partner is necessary before testing pregnant women for HIV.

### There are not enough midwives at the antenatal clinics

Majority (60.4%) of the midwives strongly agreed (25.0%) and agreed (35.4%) that there were not enough midwives at the ANCs, which implies that the midwives believed that most of the ANCs were understaffed.

### Hospitals have a shortage of HIV testing kits

Majority (70.9%) of the midwives strongly agreed (27.1%) and agreed (43.8%) that the hospitals had a shortage of HIV testing kits. This means that the midwives believed that the hospitals sometimes lack HIV testing kits. This could have a serious negative impact on PMTCT for HIV services, as HIV testing for pregnant women could be delayed. This could cause further delays when PMTCT of HIV services needs to be extended to other family members of people living with HIV.

### Attitudes of midwives towards prevention of mother-to-child transmission of HIV services

Midwives indicated that being aware of health professionals’ attitudes is critical to explain the reasons behind their level of adoption of certain health practices. Therefore, the study assessed the attitudes of midwives towards PMTCT of HIV services in the Central Region of Ghana. The results are presented in Table 3.

**TABLE 3:** Attitudes of midwives towards prevention of mother-to-child transmission of HIV services.

Attitudes	Responses	Frequency	Percentage (%)
Pregnant women should be screened for HIV.	Strongly agree	44	91.7
	Agree	4	8.3
	Disagree	0	0.0
	Strongly disagree	0	0.0
	Total	48	100.0
HIV-positive pregnant women should be referred to institutions where they can be monitored.	Strongly agree	33	68.8
	Agree	11	22.2
	Disagree	1	2.1
	Strongly disagree	3	6.3
	Total	48	100.0
HIV-infected pregnant women must deliver their babies with the aid of skilled personnel.	Strongly agree	31	64.5
	Agree	14	29.2
	Disagree	2	4.2
	Don’t know	1	2.1
	Total	48	100.0
HIV-infected women may not breastfeed their children if there is a risk of infection.	Strongly agree	18	37.5
	Agree	19	39.6
	Disagree	4	8.3
	Strongly disagree	6	12.5
	Don’t know	1	2.1
	Total	48	100.0
Pregnancy should be terminated if a mother is HIV-infected.	Strongly agree	1	2.1
	Disagree	7	14.6
	Strongly disagree	36	75.0
	Don’t know	4	8.3
	Total	48	100.0
Post-test counselling takes too much time.	Strongly agree	2	4.2
	Agree	10	20.8
	Disagree	16	33.3
	Strongly disagree	18	37.5
	Don’t know	2	4.2
	Total	48	100.0
Retesting for HIV is not necessary.	Strongly agree	3	6.3
	Agree	3	6.3
	Disagree	13	27.1
	Strongly disagree	28	58.3
	Don’t know	1	2.1
	Total	48	100.0

### Pregnant women should be screened for HIV

The midwives who strongly agreed were 91.7%, and 8.3% agreed that pregnant women should be tested for HIV. Those who disagreed and strongly disagreed were both (0%). This shows that midwives were confident about the HIV testing. For them, this would help to identify HIV-infected pregnant women to administer PMTCT of HIV services to them.

### Referral of HIV-positive pregnant women to institutions where they can be monitored

Majority of the respondents (91%) strongly agreed (68.8%) that there is a need for HIV-infected pregnant women to be referred to health facilities where they can be properly monitored. Only few of them (6.3%) strongly disagreed with this opinion, which implies that majority of the respondents had positive attitudes towards the referral of HIV-positive pregnant women to designated health facilities for critical PMTCT of HIV services.

### HIV-infected pregnant women must deliver their babies with the aid of skilled personnel

The attitudes of the respondents towards the need for HIV-infected pregnant women to deliver their babies with the aid of skilled health personnel were examined. The results showed that the majority (93.7%) of the respondents strongly agreed (64.5%) and agreed (29.2%) with this opinion.

### HIV-infected women may not breastfeed their children if there is a risk of infection

The attitudes of the respondents towards HIV-infected women breastfeeding their children if there was a risk of infection were also considered. From the results, the majority (77.1%) of the respondents strongly agreed (37.5%) and agreed (39.6%) that HIV-infected women should not breastfeed their children if there is a risk of infection, while 12.5% strongly disagreed.

### Pregnancy should be terminated if a mother is HIV-infected

Majority of the respondents (75%) strongly disagreed that pregnancy should be terminated if a mother is HIV-infected. These positive attitudes towards avoiding the termination of pregnancy indicate how confident the midwives were in the PMTCT of HIV services to ensure safe delivery for the mothers and their babies.

### Post-test counselling wastes time

From the results, majority (70.8%) of the midwives strongly disagreed (37.5%) and disagreed (33.3%) that post-test counselling wastes their time, whereas a quarter (25%) agreed. This confirms that most midwives in the region who practice PMTCT believe that post-test counselling is not a waste of time.

### Retesting for HIV is not necessary

Another attitudinal issue examined in the study was the importance of retesting for HIV in PMTCT of HIV services. It is designed to better protect infants from intergenerational transmission, prevent its spread and facilitate safe delivery with the help of skilled personnel.

Results showed that the majority (85.4%) of the respondents strongly disagreed (58.3%) and disagreed (27.1%) that retesting for HIV is not necessary. They suggest that the majority of the midwives understand and have accepted the need for retesting of HIV among pregnant women.

### Inferential analyses of perceptions and attitudes of midwives towards prevention of mother-to-child transmission of HIV services

The researchers conducted inferential analyses of various aspects of the perceptions and attitudes of midwives towards PMTCT of HIV services. The aim was to ascertain the statistical significance of various issues under investigation. In the process of conducting the inferential analyses, the researchers created composite variables for attitudes and perceptions of midwives on PMTCT of HIV services. The aim was to simplify the analysis in terms of finding relationships between the different variables and then subjecting the variables, such as attitudes and perceptions, to the various tests of differences in relation to the demographic or background data. The calculations of the composite variables were made possible for these variables because of the Likert scale measurements. In other words, the Likert-scale types of questions for attitudes and perceptions made them continuous variables, which allowed for the calculation of averages for each question under them.

In the calculation of the composite variables, ‘strongly agree’ ranged from 1.00 to 1.99, ‘agree’ ranged from 2.00 to 2.99, ‘disagree’ ranged from 3.00 to 3.99, ‘strongly disagree’ ranged from 4.00 to 4.99, while ‘don’t know’ ranged from 5.00 to 5.99.

With the composite variables, the researchers calculated the averages of each question under each category of variables (attitudes and perceptions) across the 48 sampled midwives. This enabled them to obtain the average position of the midwives on each question under the two variables. Based on the composite variables, the researchers conducted a correlation matrix to determine the nature and strength of the relationship between attitudes and perceptions of the midwives concerning PMTCT of HIV services. Correlation analysis was performed because all the variables involved were continuous or interval scale variables. The results are presented in Table 4.

**TABLE 4:** Correlation matrix for perceptions and attitudes of midwives towards prevention of mother-to-child transmission of HIV services.

Variables	Statistics	Attitudes	Perceptions
Attitudes	Pearson correlation	1.000	0.392*
	Significance (two-tailed)	-	0.006
	***N***	48	48
Perceptions	Pearson correlation	0.392*	1.000
	Significance (two-tailed)	0.006	-
	***N***	48	48

* , Correlation is significant at the 0.01 level (two-tailed).

The table also showed that there was a positive correlation between attitudes and perceptions of midwives on PMTCT of HIV services. This is reported statistically as r (statistics) = 0.392; *n* (sample) = 48; *p*-value = 0.006; *p* < 0.05. This means that as the attitudes of the midwives towards PMTCT of HIV services improved, their perceptions of PMTCT services also improved, and vice versa. The *p*-value of 0.006 showed that there was a statistically significant relationship between attitudes and perceptions of the midwives in relation to PMTCT of HIV services. This was because the *p*-value of 0.006 was within the error margin of 0.05.

## Discussion of the results

### Lack of knowledge on HIV and prevention of mother-to-child transmission services

The respondents felt that there was lack of knowledge on HIV and PMTCT services among pregnant women attending ANCs in the Central Region. This inadequate knowledge on HIV services and PMTCT among some mothers was due to the centralisation of HIV facilities in district hospitals (UNAIDS 2019:13). Thus, Ghana’s HIV treatment system and the provision of PMTCT of HIV services are concentrated in district hospitals in the district capitals. However, this has proven to be a problem for people at the subdistrict level, as some could not afford to travel regularly to district hospitals in district capitals for PMTCT of HIV services. Furthermore, health professionals in some subdistrict level health facilities did not educate mothers about these services in a timely manner. This means that efforts to decentralise HIV care and treatment services further at the subdistrict level could help raise awareness of HIV prevention and its effectiveness in treating infection-related complications.

### Less infrastructure for prevention of mother-to-child transmission services

Moreover, the perception that various designated hospitals had less infrastructure for PMTCT of HIV services was acknowledged by the majority of the midwives. According to Du et al. (2019:1), infrastructure and protective equipment are essential for the effective delivery of PMTCT of HIV services, as they contribute to the health and well-being of patients, as well as the safety of healthcare workers.

Some of these facilities could be designated areas with appropriate offices and confined areas to allow for important appointments with HIV-positive patients which could help prevent stigma, protect their identity and health status and facilitate voluntary counselling and testing. The lack of such important infrastructure could prevent some HIV-infected patient from reporting to the selected facilities for support and care. This perception could also prevent some midwives and health professionals from being fully committed to providing PMTCT of HIV services, since it could expose them to risks associated with the care and support of HIV-infected patients.

### Antiretroviral is initiated irrespective of the CD4 cell count level

The respondents admitted that ART is initiated irrespective of the CD4 cell count level. This implies that no matter the level of CD4 cell count, ART is initiated. The findings contradict with those of the studies conducted in Nigeria and Malawi whereby it was revealed that pregnant women were frequently not given ARV drugs, nor were their CD4 counts ascertained, resulting in high maternal mortality. Furthermore, the midwives did not know about the type of ARV that could be consumed by pregnant women, the transmission trajectory of HIV or the way to properly conduct a labour assessment for HIV-infected pregnant women (Meilani et al 2019:92).

Several studies from sub-Saharan Africa have highlighted significant challenges on initiating PMTCT to pregnant and postpartum women. In another study, staff attitude was noted as one of the barriers to timely care for women seeking treatment for PMTCT (Mongwenyana et al. 2020:3).

### Lack of self-care knowledge among pregnant teens

Pregnant teens lacking self-care knowledge could have negative implications for the delivery of PMTCT of HIV services to HIV-infected pregnant teens. Therefore, the midwives had to teach the teens basic self-care during pregnancy before educating them on proper PMTCT of HIV services to ensure the maximum protection for their babies. Ibrahim, Attia and Mohammed (2022:137) attest that self-care has a key role in preventing reproductive tract infection (RTI) among female adolescents. Nurses have the responsibility to educate adolescents related to various aspects about RTI and keep them free from it.

### Some religions do not accept condom use

This result indicates that some religions do not support the use of condoms, which increases the problems associated with pregnant teenage girls. According to the study conducted by Ntsime, Makhado and Sehularo (2022:4), cultural and religious beliefs make PLWH go to different churches and traditional healers for consultation and treatment. They end up not taking their treatment resulting in deterioration of health status and disease progression.

### Midwives had adequate prevention of mother-to-child transmission of HIV training

The respondents objected that the midwives lack adequate training on PMTCT of HIV to provide the needed care and support to HIV-positive patients, which implies that most of the sampled midwives had confidence in the kind of training programmes that they had received for the delivery of PMTCT of HIV services. This result contradicts the assertion by Panford (2018:43) that midwives lack training in PMTCT of HIV services and their knowledge-based on-the-job training is inadequate for administering PMTCT of HIV services. This could encourage them to provide critical PMTCT of HIV services to HIV-positive pregnant women. Although the majority of respondents were confident of the adequacy of their PMTCT training they had received, a significant proportion admitted that they lacked PMTCT of HIV training. This perception could affect service quality and midwives’ confidence in the delivery of PMTCT of HIV services in the Central Region of Ghana. This means that the provision of on-the-job training programmes on PMTCT of HIV services could help to boost the capacity of some of the health professionals in the provision of care and support to HIV-positive patients in the region.

The latter contradicts with the findings of the study conducted by Ntsime et al. (2022:8) whereby nurses and/or midwives indicated the need to be trained in the PMTCT and be motivated because, with good practice, a positive attitude and sufficient knowledge on the prevention of vertical transmission of HIV can be achieved. Rowan et al. (2018:16) alluded that training is generally considered part of any programme implementation; however, there exists little generalisable evidence that evaluates the ideal characteristics of such trainings for PMTCT services.

### Sufficient antiretroviral drugs at health facilities

The issue of no shortage of ARV drugs in the health facilities was significant in this study. This suggests that pregnant women with HIV could have an adequate amount of ARV drugs for self-treatment at home. This result also concurs with the assertion by Aboh (2018:113) that accessibility, reliability, maintainability, serviceability and securability when operationalised in health service delivery increase participation. The results also demonstrated a high level of government commitment to PMTCT of HIV services, care and treatment of PLWH, as reflected in the large budget allocations for ARV drugs in hospitals.

Tagutanazvo, Nolte and Temane (2019:6) indicated that in a study determining use and nonuse of ARVs for PMTCT in Australia, authors reported that women who participated in the study revealed that taking ARVs was quite distressing and a daily reminder of a serious, stigmatising illness. However, these same women found it helpful when they reframed the treatment as helpful to ensure an HIV-negative baby.

### Some religions do not accept visits to health facilities

The percentage of respondents that held the view that some religions did not condone visits to health facilities was alarming. This could have negative implications for PMTCT of HIV services in the region as some of the HIV-infected women may report to the health facilities only at the advanced stages of their infection. Tagutanazvo et al. (2019:6) opine that positive experiences following disclosure ranged from safe disclosure, hope for better results and hope in religion. Furthermore, one’s religious grouping contributes to promoting hope and acceptance of an HIV-positive status, thereby enabling patients to develop a positive therapeutic relationship with their ARVs and make lifestyle changes that promote adherence.

### Male partner’s consent is crucial before a pregnant woman may be tested for HIV

The need for a male partner’s approval before a pregnant woman may be tested for HIV was instrumental in the PMTCT of HIV services in the region, since the majority of the respondents agreed that such approval was necessary. This is consistent with a claim by Mongwenyana et al. (2020:3) that lack of male involvement and difficulty in disclosing results to partners by HIV-positive women were mentioned as challenging barriers to implement PMTCT programme successfully. These problems result in late initiation of ART. Lack of male partner involvement was also a known deterrent to PMTCT attendance, which was formidable challenge.

This indicates delays in the testing and counselling process during PMTCT for HIV services since some of the patients were unwilling to expose their HIV status to their spouses, while some partners too were unwilling to give their consent for their pregnant wives to test for HIV.

According to the study conducted by Chanyalew et al. (2021:1), only screening a pregnant mother is not satisfactory to PMTCT of HIV. A male partner’s involvement in HIV testing and counselling is also critical for PMTCT; however, it is one of the biggest challenges in Ethiopia.

### Insufficient midwives at the antenatal clinics

The respondents were also of the opinion that the midwives working at the ANCs in the region were understaffed, which could have serious implications for the services they provided. This supports the claim of Panford (2018:43) that shortage of midwives, combined with high workloads, could have serious implications for the management and delivery of PMTCT of HIV services, because the midwives are the same health professionals responsible for all the health problems concerning mothers.

According to Mongwenyana et al. (2020:3), in another study, service providers reported that inadequate trained personnel, workload, inadequate supply of logistics and medicines as barriers of completing the steps in the PMTCT as far as the service providers were concerned.

### Hospitals have a shortage of HIV testing kits

The respondents had the perception that the hospitals sometimes run short of HIV testing kits. This could have serious negative consequences for the PMTCT of HIV services, as there could be delays in HIV testing in pregnant women. This lack of testing kits could also be a deterrent that could further delay the extension of PMTCT services to other family members of HIV-positive persons. This concurs with the assertion of Lumbantoruan et al. (2018:7) that lack of infrastructure is one of the constraints leading to delays in the provision of PMTCT services. It should be noted that HIV testing is part of the preparatory activity in PMTCT services. As a result, anything that could cause a delay in testing could also affect other activities in PMTCT for HIV services.

However, it should be noted that any delays in determining the HIV status of pregnant women could lead to a deterioration in the health of those who may eventually test positive, as well as the health of their infants while endangering their sexual partners and other family members (Lumbantoruan et al. 2018:7).

### Pregnant women should be screened for HIV

Regarding the attitudes of the midwives towards PMTCT of HIV services, all the respondents agreed that pregnant women should be screened for HIV. This confirms their positive attitudes towards HIV screening. Mongwenyana et al. (2020:4) indicated counselling to be a facilitator for follow-up visit as well as infant testing. HIV disclosure appears to be another important facilitator and improves women’s ability to seek care at a programme for prevention of MTCT. Early presentation at facility to access care, repeat testing of HIV-negative women and uninterrupted drug supplies were main facilitators for implementation of the PMTCT. Moreover, Mongwenyana et al. (2020:11) reported that unbooked patients presented the biggest challenge during the implementation of PMTCT, for example, patients who do not know their HIV status. Some come being in labour and some refuse to be tested before they deliver.

### Referral of HIV-positive pregnant women to institutions where they could be monitored

Also, the respondents had positive attitudes towards the referral of HIV-positive pregnant women to institutions where they could be monitored. These positive attitudes were important in strengthening PMTCT of HIV services, as midwives are likely to promptly refer HIV-infected pregnant women to a well-equipped health facility which could give special attention to protect the newborns from the infection. Oleribe et al. (2018:258) posited that the early access to PMTCT of HIV services for HIV-positive pregnant women is important to protect infants from getting the infection and an important step in preventing generational transmission of HIV.

### Delivering with the aid of skilled personnel

The respondents agreed that HIV-infected pregnant women’s babies must be delivered by skilled personnel. Anaba, Ukwenga and Sam-Agudu (2018:1) in their study reported that delivering babies to HIV-positive pregnant women by skilled healthcare workers with prior knowledge of the mother’s HIV status allows healthcare professionals to follow strict protocols to protect babies from getting the infection, while preventing accidental infection by midwives. Anaba et al. (2018:1) posited that measures to prevent MTCT of HIV and to protect health professionals from accidental infections are critical in the PMTCT of HIV services to curb the spread and transmission of the infection. This is the reason why the Ghana Health Service has made HIV screening part of the safety protocols in antenatal services. As such, this knowledge enables healthcare professionals to provide critical care services to improve safe delivery. The positive attitudes of most of the midwives towards the need for HIV-infected pregnant women’s babies to be delivered by skilled health professionals are therefore an attestation of the high level of understanding and acceptability of the need to ensure optimum protection for babies and birth attendants. In other words, health professionals and infants could be at a greater risk of contracting the virus as a result of poor attitudes towards PMTCT of HIV service protocols.

The motivation for positive attitudes towards safety protocols is partly informed by their knowledge of the risk of exposure, the severity of the threat of breaking safety protocols and their capacity to cope with contracting diseases associated with their exposures (Littlewood & Greenfield 2018:5).

### HIV-infected women should not breastfeed their children if there is a risk of infection

The study revealed that HIV-infected women should not breastfeed their children if there is a risk of infection. The consensus of most of the midwives about breastfeeding of infants born to HIV-infected mothers could enable them to educate such mothers to use alternative means of feeding their babies to prevent MTCT. The Center for Disease Control and Prevention (CDC 2020:1) of the United States stated that breastfeeding significantly increases the risk of MTCT of HIV.

### Pregnancy should not be terminated if a mother is HIV-infected

From the study, the respondents had positive attitudes towards allowing HIV-infected mothers to carry their pregnancies without terminating it. Thus, most of the midwives had confidence in their skills and systems in providing critical PMTCT of HIV services to prevent further transmission from HIV-infected pregnant women to their babies through pregnancy, delivery and postpartum services. This great consensus among the midwives about permitting HIV-positive pregnant women to carry their pregnancy to term could be attributed to the structures, systems and protocols put in place by Ghanaian Health. Mongwenyana et al. (2020:14) alluded that most notably, both healthcare providers and patients understood the significance of implementing PMTCT for maternal health reasons and prevent HIV transmission.

### Post-test counselling is not a waste of time

The results showed that the midwives had positive attitudes towards post-test counselling in the PMTCT of HIV services. This was important because, according to Meilani et al. (2019:92), post-test counselling helps to manage the shock, anxiety, fear and misconceptions surrounding the infection. This contrasts with the assertion by Mongwenyana et al. (2020:12) that patients do not have adequate time to process the information surrounding their HIV status and do not understand what is been explained to them. Furthermore, healthcare providers explained that there is a challenge with testing the exposed babies because sometimes they do not have polymerase chain reaction (PCR) kits at facilities while some patients refuse to test their babies after delivery.

### Retesting for HIV is necessary

The majority of midwives agreed that retesting for HIV is necessary. This was important because retesting for HIV during pregnancy allows both mother and baby to receive early care to prevent further transmission (Mandala et al. 2019:4).

Tian et al. (2023:10) attest that the prevalence of infections among infants and children under 5 years of age is high. The rates of MTCT remain high despite effective prevention measures. Several pregnant women living with HIV and AIDS cannot obtain adequate testing and treatment. Thus, it coincided with the study conducted by Meilani et al. (2019:91) whereby most of the midwives indicated that as part of PMTC, they counselled pregnant women to take the HIV test. They also offered informed consent and performed the counselling after the test.

### Strengths and limitations of the study

Exploring midwives’ perceptions and attitudes on PMTCT of HIV services will help promote much awareness of the services, reduce MTCT of HIV and extend the life of mothers and their children in the Central Region and Ghana at large. It is expected that the use of longitudinal studies could have provided programme implementers with a suitable platform to collect more data for a clearer assessment of PMTCT of HIV services. However, time and financial constraints prevented the researchers from following this approach. To mitigate this limitation, the researchers asked questions that enabled the respondents to reflect on their past experiences in the programme before providing their conclusions.

At the time of data collection, the COVID-19 pandemic in Ghana delayed the data collection process as most of the district hospitals denied us access to their facilities. This also affected the amount of time spent with each respondent, since the more time spent with someone or outside of the home, the greater the risk of contracting COVID-19. As a result, in-depth communication with the respondents was hampered. However, the researchers were able to adhere to the COVID-19 protocol, which included the use of personal protective equipment necessary for a successful data collection process.

## Recommendations

The Ghana Health Service to:

- Launch a massive campaign on PMTCT of HIV services to raise public awareness. This could be done through media programmes to educate the public about the tenets of PMTCT of HIV services.
- Manage its stocks of HIV testing kits effectively to avoid shortages of these kits at the various health facilities. This is even more important because of the researchers’ recommendation that these health facilities be used more often in the provision of PMTCT of HIIV services.
- Intensify the provision of free infant formula to HIV-positive mothers to supplement their infant feeding. In addition, they recommend that the midwives provide more education on the alternative local feeding formulas to enable HIV-positive lactating mothers to avoid breastfeeding their infants.
- Use a qualitative approach in the future so that HCPs and midwives can freely express their perceptions and experiences other than being limited by having to choose from the survey questionnaire can elicit more that can be used to improve the PMTCT programme.

## Conclusion

Prevention of mother-to-child transmission of HIV services has become an integral part of antenatal services to ensure effective HIV control in pregnant women and to prevent or reduce transmission of HIV to their children. A major issue in strengthening PMTCT services among antenatal attendees is promoting awareness.

In this study, the researchers aimed to explore and describe the perceptions and attitudes of midwives towards PMTCT of HIV services. The study found that the midwives generally had positive perceptions and attitudes towards PMTCT of HIV services because of their many years of experience in providing these services to antenatal attendees. This means that the midwives’ level of experience was critical in improving their perceptions of PMTCT of HIV services. As a result, midwives were generally committed in ensuring that the pregnant women adhered to the various protocols of PMTCT of HIV services.

Part of promoting an awareness of PMTCT of HIV services by the midwives involves providing pre- and post-test counselling to pregnant women, providing a user-friendly environment for pregnant women and encouraging the participation of the male partners of pregnant women in PMTCT of HIV services.
